# Nurses’ strategies for overcoming barriers to fundamental nursing care in patients with COVID‐19 caused by infection with the SARS‐COV‐2 virus: Results from the ‘COVID‐NURSE’ survey

**DOI:** 10.1111/jan.15261

**Published:** 2022-04-25

**Authors:** Holly V. R. Sugg, David A. Richards, Anne‐Marie Russell, Sarah Burnett, Emma J. Cockcroft, Jo Thompson Coon, Susanne Cruickshank, Faye E. Doris, Harriet A. Hunt, Heather Iles‐Smith, Merryn Kent, Philippa A. Logan, Leila M. Morgan, Naomi Morley, Anne Marie Rafferty, Maggie H. Shepherd, Sally J. Singh, Susannah J. Tooze, Rebecca Whear

**Affiliations:** ^1^ College of Medicine and Health University of Exeter Exeter UK; ^2^ Department of Health and Caring Sciences Western Norway University of Applied Sciences Bergen Norway; ^3^ The National Institute for Health Research (NIHR) Applied Research Collaboration (ARC) South West Peninsula (PenARC) Exeter UK; ^4^ The Royal Marsden NHS Foundation Trust London UK; ^5^ School of Health and Society University of Salford Salford UK; ^6^ Northern Care Alliance NHS Group Salford UK; ^7^ School of Medicine University of Nottingham Queens Medical centre Nottingham UK; ^8^ Faculty of Nursing Midwifery and Palliative Care King's College London London UK; ^9^ NIHR Exeter Clinical Research Facility Royal Devon and Exeter NHS Foundation Trust Exeter UK; ^10^ Department of Respiratory Science University of Leicester Leicester UK; ^11^ University Hospitals of Leicester NHS Trust Biomedical Research Centre – Respiratory Glenfield Hospital Leicester UK

**Keywords:** COVID‐19, fundamental nursing care, nurses, nursing interventions, qualitative, SARS‐CoV‐2, survey

## Abstract

**Aims:**

To identify strategies used by registered nurses and non‐registered nursing care staff in overcoming barriers when providing fundamental nursing care for non‐invasively ventilated inpatients with COVID‐19.

**Design:**

Online survey with open‐ended questions to collect qualitative data.

**Methods:**

In August 2020, we asked UK‐based nursing staff to describe any strategies they employed to overcome barriers to delivering care in 15 fundamental nursing care categories when providing care to non‐invasively ventilated patients with COVID‐19. We analysed data using Framework Analysis.

**Results:**

A total of 1062 nurses consented to participate in our survey. We derived four themes. 1) Communication behaviours included adapting verbal and non‐verbal communication with patients, using information technology to enable patients’ significant others to communicate with staff and patients, and establishing clear information‐sharing methods with other staff. 2) Organizing care required clustering interventions, carefully managing supplies, encouraging patient self‐care and using ‘runners’ and interdisciplinary input. 3) Addressing patients’ well‐being and values required spending time with patients, acting *in loco familiae*, providing access to psychological and spiritual support, obtaining information about patients’ wishes early on and providing privacy and comforting/meaningful items. 4) Management and leadership behaviours included training, timely provision of pandemic information, psychological support, team huddles and facilitating regular breaks.

**Conclusions:**

Our respondents identified multiple strategies in four main areas of clinical practice. Management and leadership are crucial to both fundamental care delivery and the well‐being of nurses during pandemics. Grouping strategies into these areas of action may assist nurses and leaders to prepare for pandemic nursing.

**Impact:**

As these strategies are unlikely to be exclusive to the COVID‐19 pandemic, their global dissemination may improve patient experience and help nurses deliver fundamental care when planning pandemic nursing. However, their effectiveness is unknown. Therefore, we are currently evaluating these strategies in a cluster randomized controlled trial.

## 1 INTRODUCTION

The challenges of the SARS‐COV‐2 viral pandemic and the disease it causes (COVID‐19) have led to an increase in missed nursing care or ‘care left undone’, defined as any aspect of nursing care that is omitted or delayed, in part or in whole (Kalisch et al., 2009). In our survey of 1062 UK nursing staff caring for inpatients with COVID‐19, respondents rated their ability to meet patients’ needs in many fundamental areas of nursing care as worse for hospitalized patients with COVID‐19 than for other patients (Sugg et al., 2021), acknowledging that care for these patients was being missed more frequently than in other clinical situations. In a parallel systematic review of 64 articles on fundamental nursing care in five pandemics, we synthesized reports on nursing strategies used to address barriers to care (Whear et al., 2022), but found that the literature was mostly non‐empirical, of poor quality and provided little robust guidance to nurses. To design and test a fundamental care nursing protocol for non‐invasively ventilated inpatients with COVID‐19 (Richards et al., 2021), we also asked nurses experienced in caring for these patients what they did to reduce the potential for missed care. We report that data here.

## 2 BACKGROUND

Patient experience of care is associated with safety, clinical effectiveness, care quality, treatment outcomes, costs and service use (Black et al., 2014; Darzi, 2008; Doyle et al., 2013). Nursing care is a key determinant of this experience (Graham et al., 2018, Murrells et al., 2013). Patient safety failures are correlated with a high prevalence of missed nursing care (Aiken et al., 2014; Aiken et al., 2017; Ausserhofer et al., 2014; Kalisch, 2006; Kalisch et al., 2009). The extent of missed care is related to poor patient outcomes, increased mortality and adverse events and poor patient satisfaction and experience (Ball et al., 2018; Bruyneel et al., 2015; Kalisch et al., 2014; Recio‐Saucedo et al., 2018).

Both nurses and patients have indicated that important elements of care are regularly missed, including nutrition, hygiene (e.g. bathing; mouth care), ambulation/supporting mobility, communication/talking with patients and emotional and psychological support (Ball et al., 2016; Griffiths et al., 2018; Kalisch, 2006; Kalisch et al., 2014). These elements are regarded as ‘fundamental’ care: ‘actions on the part of the nurse that respect and focus on a person's essential needs to ensure their physical and psychosocial wellbeing’ (Feo et al., 2018, p. 2292). These needs are met by developing a positive and trusting relationship with the patient and their family/carers; thus, these actions include nurses’ relational as well as transactional behaviours (Feo et al., 2018; International Learning Collaborative, 2019).

We undertook our survey in the early phase of the pandemic in the UK during a time that information and understanding of the virus were lacking and vaccines were not available (Sugg et al., 2021). Healthcare services were disrupted by very large numbers of patients admitted who were being cared for by nurses and other care staff unfamiliar with pandemic nursing (Maben & Bridges, 2020). The majority of our respondents rated care as worse for COVID‐19 patients than others they cared for in specific areas of mobility, talking and listening, non‐verbal communication, communicating with relatives, carers and significant others and caring for patient's emotional well‐being, anxiety and depression. In all other areas of care, around one‐third of respondents indicated that care was also worse for these patients (Sugg et al., 2021).

Our respondents identified specific barriers to meeting patients' fundamental care needs. Foremost was infection control, specifically nursing patients in isolation and wearing personal protective equipment. Insufficient stock, and staffs' inability to take items in and out of isolation rooms without donning and doffing personal protective equipment, also impeded physical care. Another significant barrier was the lack of presence from specialist services and a lack of expertise in redeployed staff themselves. Time, or the lack of it, prevented respondents from talking and listening to patients, although another highly cited barrier was the staffs’ own reluctance to spend time with patients for fear of catching COVID‐19. This was compounded by a lack of knowledge about COVID‐19 which impeded respondents’ ability to answer patients’ questions. Throughout their accounts, respondents also highlighted the impact of restrictions on visitors for both patients and nursing staff (Sugg et al., 2021).

Inpatients with COVID‐19 have also reported experiencing poor communication, a lack of support and assistance, insufficient information and/or equipment (Healthwatch, 2020) together with a range of negative emotions and psychological consequences due to a lack of social interaction, limited mobility, difficulties communicating with staff and a lack of information about their illness and treatment (Shaban et al., 2020). In addition, patients’ relatives, carers and significant others have reported poor communication from staff and may not be kept well informed about the patient (Healthwatch, 2020).

These reports mirror findings from our own systematic review on fundamental nursing care in pandemic situations, where we identified wearing personal protective equipment, adequate staffing, infection control procedures and emotional challenges as barriers to care (Whear et al., 2022). Our review demonstrated the poor quality of research on pandemic nursing practice with only 19 empirical articles in 64 included papers covering five pandemics. Despite the substantial barriers to care identified, at the time of undertaking this review and our survey, there were no evidence‐based guidelines for nursing patients infected with the SAR‐COV‐2 virus who are not invasively ventilated (i.e. may be receiving no ventilation or ventilatory support without tracheal intubation), who represent the majority of hospitalized patients under this condition (Torjesen, 2021).

Consequently, we designed the COVID‐NURSE fundamental nursing care clinical protocol for patients hospitalized with COVID‐19 not invasively ventilated for evaluation in a cluster randomized controlled trial (Richards et al., 2021) using data from i) a survey of nurses’ experiences of caring for patients with COVID‐19, including the barriers encountered in delivering fundamental care (reported elsewhere, Sugg et al., 2021) and strategies adopted to overcome these (reported here); ii) a systematic review (Whear et al., 2022); and iii) co‐creation workshops involving patients with experience of hospitalization with COVID‐19 and nurses caring for them.

In this paper, we report our survey data on strategies used by nurses to overcome barriers to care. We describe our study using both cross‐sectional (STROBE) (Knottnerus & Tugwell, 2008) and qualitative (COREQ) (Tong et al., 2007) study reporting guidelines.

## 3 THE STUDY

### 3.1 Aim

To identify strategies used by registered nurses and non‐registered nursing care staff in overcoming barriers when providing fundamental nursing care for non‐invasively ventilated inpatients with COVID‐19.

### 3.2 Design

We undertook a qualitative study with data collected using open‐ended survey questions and analysed using a Framework approach (Ritchie et al., 2013).

### 3.3 Setting

We collected data for the study in the UK using Qualtrics™ online survey software (Qualtrics, 2020).

### 3.4 Participants

Eligible respondents were UK‐based registered nurses and non‐registered auxiliary nursing/healthcare support workers/assistants working in geographically diverse general or specialist hospitals, who had the experience of nursing non‐invasively ventilated inpatients with COVID‐19. Respondents who had only nursed invasively ventilated patients were ineligible.

### 3.5 Survey instrument

We structured our survey according to the Fundamentals of Care model (Feo et al., 2018; Kitson et al., 2010), including sections on physical, relational and psychosocial care areas, with subsections in each area corresponding to sub‐categories of care adapted from Feo et al. (2018)) (Table 1). In each subsection, we asked respondents to provide free text describing what, if any, strategies they employed to try and overcome barriers to that sub‐category of care. We also collected demographic information (Table 2). In developing the survey, we sought advice from members of our patient and public involvement group, our patient and public involvement co‐investigator, the COVID‐NURSE trial co‐investigators and members of the wider research team. We piloted the survey with four nursing teams and amended it according to their feedback.

**TABLE 1:** Survey structure: Fundamental care areas and sub‐categories of care

Section: Care area	Subsection: Sub‐category of care
1. Physical	1. Hygiene, personal cleansing and toileting
	2. Eating and drinking
	3. Rest and sleep
	4. Mobility
	5. Patient comfort
	6. Patient safety
	7. Medication management
2. Relational	1. Establishing a relationship with patients
	2. Talking and listening
	3. Non‐verbal communication
	4. Shared decision‐making
	5. Communicating with relatives, carers and significant others
3. Psychosocial	1. Dignity and respect
	2. Respecting patients' values and beliefs
	3. Well‐being, anxiety and depression

**TABLE 2:** Respondent characteristics

Gender	Female	858 (87.7)
	Male	112 (11.5)
	Prefer not to say	8 (0.8)
Age	<25	98 (10.0)
	26–30	173 (17.7)
	31–40	257 (26.3)
	41–50	234 (23.9)
	51–60	182 (18.6)
	61–66	26 (2.7)
	>67	1 (0.1)
	Prefer not to say	7 (0.7)
Ethnicity	Asian/Asian British	32 (3.3)
	Black/African/Caribbean/ Black British	15 (1.5)
	Mixed/Multiple ethnic groups	13 (1.3)
	Other ethnic groups	46 (4.7)
	Other White	85 (8.7)
	White British	779 (79.7)
	Prefer not to say	8 (0.8)
Environment	Acute General NHS hospital including teaching hospital	898 (91.8)
	Tertiary/specialist	63 (6.4)
	Private healthcare	6 (0.6)
	Missing data	11 (1.1)
Country	England	933 (95.4)
	Wales	15 (1.5)
	Scotland	5 (0.5)
	Northern Ireland	4 (0.4)
	Other country	1 (0.1)
	Missing data	20 (2.0)
Main position	Charge nurse	206 (21.1)
	Staff nurse	374 (38.2)
	Specialist/advanced nurse	142 (14.5)
	Research nurse	42 (4.3)
	Nurse researcher	1 (0.1)
	Manager	73 (7.5)
	Student nurse	20 (2.0)
	Non‐registered nursing associate	10 (1.0)
	Non‐registered care or nursing assistant	90 (9.2)
	Missing data	20 (2.0)
Redeployed?	Yes	139 (14.2)
	No	227 (23.2)
	Missing data	612 (62.6)
Usually work on respiratory ward?	Yes	114 (11.7)
	No	252 (25.8)
	Missing data	612 (62.6)
Usually work in non‐ward environment?	Yes	138 (14.1)
	No	228 (23.3)
	Missing data	612 (62.6)

Data are number (%); percentages may not always total 100 due to rounding.

### 3.6 Data collection

We ran the survey from the 3rd to 26th August 2020. As our study was undertaken rapidly to provide information to guide our ‘COVID‐NURSE’ (Richards et al., 2021) intervention development, our sample size was not predetermined, seeking to recruit as many respondents as possible during the survey timeframe. We invited a convenience sample of respondents by circulating the survey link to a database of nurses who had consented to be approached for COVID‐19‐related research studies through the ‘Impact of COVID‐19 on the Nursing and midwifery workforce’ (ICON) study (University of Cardiff, 2021); networks of senior research, management and clinical nurses in England and Wales including the National Institute for Health Research 70@70 research network, the Association of UK Lead Research Nurses, the Royal Colleges and hospital sites affiliated with the COVID‐NURSE Trial co‐investigators; the UK National Institute for Health Research Clinical Research Network and through social media including Twitter and University of Exeter channels.

We sent a survey link to nurses on our database and to key gatekeepers in the networks listed above. We asked gatekeepers to circulate the link via newsletters, emails and other communication channels appropriate to their networks with a covering letter informing potential respondents of the purpose and timeframe for the survey. The landing page for the survey provided links to the participant information sheet, frequently asked questions and the survey.

### 3.7 Ethics considerations

We were granted ethics approval by the University Ethics Committee (Application Number Jul20/D/256) and the United Kingdom Health Research Authority research and development governance assurance (IRAS reference 287288). At the beginning of the survey, respondents electronically gave their informed consent to participate, which included consent to publish anonymized respondent data.

### 3.8 Data analysis

The UK Clinical Research Collaboration (UKCRC)‐registered University of Exeter Clinical Trials Unit received, cleaned and processed the data. Study researchers uploaded anonymized data sets to Microsoft Excel (Microsoft Coproration, 2013). We applied pairwise deletion to each survey item to account for missing data and maximize the data available for analysis. For demographic variables, we calculated percentages from the number of respondents who provided data for that specific variable. We combined ethnicity data into standard categories (Office for National Statistics, 2019).

We analysed data using Framework Analysis (Ritchie et al., 2013) to allow for both inductive and deductive approaches in combining our study aims/survey questions with respondents’ original accounts (Pope & Mays, 2006; Ritchie et al., 2013).

We achieved familiarization with the data through reading responses and then completed the first cycle of coding of responses, developing an initial thematic framework as we analysed batches of surveys (Miles et al., 2014). Using this framework, we completed second cycle coding and analysed responses thematically using a constant comparison approach, examining similarities and differences to categorize the strategies described in our framework (Miles et al., 2014; Thorne, 2000).

We organized survey responses by sub‐category of care (Table 1). We analysed all responses (regardless of sub‐category of care) together rather than analysing each sub‐category independently; however, we maintained a record of which sub‐category they related to in order to inform our interpretation of the data and understanding of context and meaning. We thus charted data in an analytic/framework matrix to allow analysis in each theme, as well as across each sub‐category of care, and the further refinement of themes (Ritchie et al., 2013, Miles & Huberman, 1994, Spencer et al, 2014).

### 3.9 Validity and reliability/rigour

We were a multidisciplinary team consisting of four researchers (HH, DR, AMRu, HS) of mixed gender with backgrounds in nursing (2), nursing education (2), mental health services research (2) and clinical research (3). All researchers have PhDs, were trained and experienced in qualitative data analysis and were employed as a research fellow (HH), Lecturer (HS), Senior Lecturer (AMRu) or Professor (DR) at the time of the study. AMRu independently coded a subset of the raw data which HS double‐coded and verified, and the research team discussed the development of themes until consensus could be achieved, supporting the credibility and reliability of data interpretation (Barbour, 2001; Ritchie et al., 2013). Thus, the rigour of our approach and validity of our analysis were enhanced through the consideration of multiple differing perspectives in our research team (Barbour, 2001; Houghton et al., 2013; Ritchie et al., 2013) alongside our researchers’ expertise and skills in the methods used (Yardley, 2017).

### 3.10 Patient and public involvement

When designing the survey and all other aspects of the project, we were advised by a Patient Advisory Group consisting of eight people who had experience of being cared for in hospital with COVID‐19 or were a relative of someone who had been admitted.

## 4 FINDINGS

A total of 1062 eligible respondents consented to provide survey data; 84 of these provided no further data. Respondent characteristics are summarized in Table 2.

The number of respondents specifically providing narrative data on strategies for each sub‐category of care ranged from 46 to 318 (Table 3).

**TABLE 3:** Number of respondents providing data for each survey item

Care area	Sub‐category of care	Number respondents
1. Physical	1. Hygiene, personal cleansing and toileting	318
	2. Eating and drinking	202
	3. Rest and sleep	146
	4. Mobility	131
	5. Patient comfort	128
	6. Patient safety	115
	7. Medication management	70
2. Relational	1. Establishing a relationship with patients	143
	2. Talking and listening	91
	3. Non‐verbal communication	70
	4. Shared decision‐making	53
	5. Communicating with relatives, carers and significant others	137
3. Psychosocial	1. Dignity and respect	60
	2. Respecting patients' values and beliefs	46
	3. Well‐being, anxiety and depression	67

The strategies described by respondents were understood in four themes as follows: (1) Communication behaviours, (2) Organization of care, (3) Addressing patients’ well‐being and values, (4) Management and leadership. Each theme encompassed several constituent themes. Respondent ID numbers are provided in brackets after quotations.

### 4.1 Theme 1: Communication behaviours

Respondents described adapting communication methods with patients (constituent theme a), patients’ significant others (constituent theme b) and other staff (constituent theme d), alongside strategies to help patients communicate with their significant others (constituent theme c). These adaptations were highlighted in the context of personal protective equipment presenting a significant barrier to relational care, compromising hearing, lip reading, facial expressions, non‐verbal cues and patients’ recognition of staff. Strategies also included responses to restrictions on visitors, which impeded communication with them and impacted patients’ well‐being.

#### 4.1.1 Nurse–patient communication

Largely due to the challenges of recognizing speech and expressions through personal protective equipment, respondents described increasing non‐verbal communication with patients. This included touch, hand‐holding, writing information, using pictures, gesticulation and body language.

Because masks are now used all the time, you find yourself using different ways to communicate. More written communication, or signing, using pictures. (ID111)

Hand holding; reassuring touch to their arm, shoulder; nodding of [the] head to show understanding; we used whiteboards to write things down. (ID627)

Respondents also described increasing eye contact and ‘using your eyes to “communicate”’ (ID171):

I tried to ensure that my smile reached my eyes because they couldn't see my smile through my mask. (ID80).

Similarly, respondents highlighted changing verbal communication to help patients hear them (‘I tried my best to enunciate’ (ID475); ‘we had to speak in clear, concise words’ (ID73)) and to vocalize their facial expressions:

It is surprising how you can use your eyes to say things ‐ but it isn't the same ‐ we had to tell people our emotion – i.e. ‘I am smiling’. (ID421)

Most nurses experienced the pandemic as a novel clinical situation, including factors like wearing personal protective equipment, and patients' fears. Respondents described an increased need to provide information and explanation to patients. In particular, respondents ‘told the patient which member of staff was with them and told them what [they] were going to do before [they] did anything’ (ID140), ‘explained to each patient why personal protective equipment was being worn’ (ID79) and tried to keep patients ‘up to date with the latest evidence and ever changing environment’ (ID599).

In the context of personal protective equipment, respondents emphasized the need to clarify their identity at each interaction with patients, and typically ‘created and wore face name badges so patients knew what we looked like outside of wearing the mask’ (ID229). Given the increased reliance on verbal communication, respondents also took special care to ensure patient's understanding, for example ‘restating what understanding there was of information given and received’ (ID315).

#### 4.1.2 Nurse‐significant other communication

Restrictions on face‐to‐face visits changed how respondents communicated with patients' significant others for both updating them and obtaining information. Respondents highlighted the need for regular (at least daily), scheduled telephone or video contact by a named staff member, ideally in a dedicated ‘family liaison team’ and/or ‘information hub’, to prevent all staff from regularly donning and doffing personal protective equipment.

Respondents noted the need to ensure safety and confidentiality during remote communication; for example staff may only communicate with one named next of kin to reduce sharing of patient information. Respondents typically reported using a password‐protected system:

We asked patients if we could share information with their relatives, then set up passwords so we could identify them. We gave more information over the phone than we would normally give. (ID141)

Respondents reported documenting communication with significant others, for example on a ‘specific communication form’ (ID234), so that all staff were aware of the communication held and could implement any requests:

[We] encouraged doctors and nurses to document when they had spoken to relatives and what was said. (ID229).

#### 4.1.3 Patient‐significant other communication

Key to addressing visitor restrictions was using technology (telephone and/or video calls) for ‘virtual visiting’ between patients and their significant others, in a regular and planned manner, potentially using a booking system:

Patients were helped to keep in communication with their loved ones by telephone or video calls. Nurses helped patients to download WhatsApp to their personal phone and to use it for the first time. Where this was not possible the [hospital] quickly implemented tablet computers on each ward with a booking system for relatives to call their loved one. (ID234)

Thus, respondents often needed to assist patients with using technology, and ward phones, tablets and/or smartphones were often provided:

We asked for [a] cordless ward phone as the area we were in didn't have bedside phones and we also got set up with video calling on an iPad so patients could still have the human contact from their loved ones which they appreciated. (ID349)

#### 4.1.4 Staff communication

Respondents adapted their communication with each other, and the interdisciplinary team, to share information effectively while adhering to infection control procedures. Key to this was reducing the number of staff entering a patient's room by displaying patient information either inside rooms where it was visible outside, outside rooms or outside the ward:

We have drywipe white boards on the outside of the cubicles upon which we can write any relevant info. (ID256).

[We had] signs outside of [the] covid area detailing dietary requirements of patients. (ID238)

This particularly facilitated communication with other staff such as catering staff. Other methods for staff communication across different areas included using iPads and/or walkie‐talkies, particularly when requesting supplies.

Respondents also noted the importance of keeping clear, detailed and visible records of patients' needs and care received:

[We] completed a daily chart indicating each time [the] patient was turned, skin checked etc…, ensured patients were on a food chart, care plans updated and notes documented, concerns handed over during hand over, white board updated with the nutritional status, magnets used behind patients beds to highlight their diet requirements. (ID536)

### 4.2 Theme 2: Organization of care

Respondents described their (re‐)organization of care, particularly in terms of clustering interventions (constituent theme a), managing supplies (constituent theme b), encouraging self‐care (constituent theme c) and teamwork (constituent theme d). These strategies were typically used to meet patients' physical care needs effectively and efficiently, in the context of infection control procedures, insufficient stock, staffs' inability to take items in and out of isolation rooms without donning and doffing personal protective equipment, and reduced interdisciplinary presence.

#### 4.2.1 Clustering interventions

To maintain infection control procedures, reduce donning and doffing of personal protective equipment, ‘minimise the number of times [staff] were exposed to the patient’ (ID2261) and ‘avoid waking someone up multiple times’ (ID558), respondents described clustering interventions: completing as many nursing interventions as possible in one visit to a patient.

We tried to reduce the amount of times we had to go in to patients, doing numerous care things on each visit: personal care, meals, toileting, drinks, bed changing, but still maintaining dignity and obviously not refusing to do these things if required at other times. (ID368)

This involved being aware of all of the patients' potential needs and enquiring into these to ensure they were not missed. Respondents particularly highlighted offering a drink at every opportunity and coordinating activities such as mouthcare (ID340) and taking meal orders (ID328) with more scheduled tasks such as providing meals and turning patients.

Encouraging fluid intake (tea, coffee, etc) whenever entering patient's room… if bringing [the] patient some water, also asking if they need the toilet and bringing any medications they may need. (ID516)

Respondents described the importance of explaining to patients that they were grouping activities, and why, to help encourage patients to ensure that all of their own needs had been met. Respondents also noted one danger associated with this approach, in that patients saw staff less regularly:

This did increase the overall care of the patient but also meant there was reduced overall contact. (ID85)

Thus, this needs to be balanced with strategies to meet patients' psychosocial needs and circumvent their social isolation.

#### 4.2.2 Managing supplies

Connected to clustering interventions was managing supplies to ensure that staff took everything they needed into a patient's room:

[You] made sure that if you were going inside a patient's room and about to put on personal protective equipment, that you had everything beforehand. (ID228)

Some respondents ‘developed a system’ (ID16) or ‘created a planned checklist’ (ID33) to assist with this and ensured that supplies were kept in accessible places either inside or outside patients' rooms:

We created trolleys outside rooms with supplies on, e.g. wipes, bags, linen etc…to prompt people to take everything in. (ID558)

Respondents also reported using broader stock management strategies, including regular re‐stocking checks and ensuring sufficient supplies to items and equipment, such as hoists and commodes, were regularly ordered and made available:

Daily checking and ordering [of] disposable cups, plates, bowls, utensils from kitchen. Ordering bottled water. (ID587)

#### 4.2.3 Encouraging self‐care

To assist in meeting patients' physical care needs, respondents encouraged patients to meet their own needs where possible, ‘do whatever they could for themselves’ (ID334), and ‘maintain their independence as much as possible to prevent dehydration and lack of nutritional intake’ (ID512). Respondents also encouraged patients to maintain their hygiene, and approach rest effectively:

Promoting sleep hygiene to patients using their phones before bed. Encouraging patients to not sleep too much during the day. (ID243)

To support patients’ mobility (a significant area of missed care for patients with COVID‐19, Sugg et al., 2021), in the absence of patients being able to mobilize around the ward, respondents encouraged patients to mobilize in their rooms. In particular, respondents provided exercise advice and encouraged walking around the room, leg exercises such as squats, bed exercises and sitting out of bed/in chairs.

I ensured if medically able that all patients were sat out of bed for set periods of time and were taught chair exercises. (ID491)

#### 4.2.4 Teamwork

Connected to clustering interventions and managing supplies were ‘runners’ or buddy systems: respondents worked in pairs to manage patients' needs efficiently, and fetch supplies while maintaining infection control procedures:

[We had] a "buddy" system in place so someone not in personal protective equipment/contact with the patient would be a "runner" to retrieve things. (ID492)

We had a clean and dirty nurse outside and inside the bay to pass through equipment to avoid donning and doffing and wastage of personal protective equipment. (ID493)

Respondents also considered multidisciplinary working and support from interdisciplinary colleagues (e.g. physiotherapists, counsellors, dieticians, pharmacists, chaplains) as crucial to meeting patients’ needs. In the context of reduced presence from interdisciplinary staff, respondents highlighted the importance of establishing regular, scheduled interdisciplinary liaisons, and some described measures taken to improve interdisciplinary input:

There was a 'proning' team made up of redeployed surgeons and other theatre staff who regularly came around to turn proned patients at set times. (ID427)

Our pharmacists changed to a shift working pattern which provided seven day cover, pre‐COVID we didn't have ward based pharmacy coverage at weekends. (ID321)

Lots of physio and occupational therapy staff were redeployed… This meant we had more support and gave staff more confidence to mobilise patients. (ID458)

Respondents also noted specific methods of communicating with other staff such as ‘identification outside of the bays of patients' specific dietary needs’ (ID626) and creating ‘an emailing system for dieticians to email feed schedules’ (ID1585).

### 4.3 Theme 3: Addressing patients’ well‐being and values

Respondents described strategies to maintain and respect patients’ views, dignity and well‐being, including supporting mental health (constituent theme a), planning ahead with patients (constituent theme b) and providing privacy, comfort and meaning (constituent theme c). These were highlighted particularly in relation to deficits in meeting patients’ psychosocial needs, especially in terms of their emotional well‐being, anxiety and depression. This was in the context of visitor restrictions, patients’ fear and anxiety around COVID‐19, personal protective equipment impeding relationship‐building with staff and the severity of patients’ conditions compromising their participation in decision‐making.

#### 4.3.1 Supporting patients' mental health

Respondents described supporting patients’ well‐being by spending time with, talking to, and reassuring patients. This included enquiring about their wishes, families, interests, how they were feeling and whether anything could be done for them; explaining why things were happening (e.g. ‘what's going on, why everybody wears personal protective equipment, where their families are’ [ID355]) and tending to their spiritual needs:

[I] tried to spend time with patients… Being there for patients who were taking their last breath, holding their hand and trying to make their death as personalised as possible for them. For some I found out if they were religious or not and prayed with them if they were. If not, I would find their favourite music and play it for them. (ID40)

Related to this were respondents' attitudes of *in loco familiae*: that is acting in the place of family by understanding patients' individual needs and providing things which significant others normally would, such as advocating for patients and providing snacks:

I urged my team to think about the 'Covid patients' still as patients and to imagine how scared they were and how we [would] want to treat them if they were our relatives. (ID15)

If there were any requests from family… I would try and do what they wanted for their loved one due to them not being able to visit. (ID268)

Some respondents described having ‘bedside buddies’ (such as healthcare assistants) to talk with patients, and tend to these needs:

Bedside buddies helped with emotional wellbeing, anxiety and low mood as I have seen them provide these needed talks with patients, sometimes just taking their mind off of things helped them elevate their mood. (ID73)

Also described was access to counsellors, psychology staff and chaplains, whether face‐to‐face or remote, as solutions to supporting patients' mental health:

Patients were offered counselling sessions by phone/video call. (ID458)

[The] chaplaincy team worked very hard to provide spiritual support to patients. Some of this was done virtually and some will be the chaplaincy wearing personal protective equipment. (ID455)

#### 4.3.2 Planning ahead with patients

To ensure that patients' wishes and beliefs were respected in emergency or end of life decision‐making, respondents noted the importance of obtaining this information on admission or from significant others:

We identify cultural beliefs and values at their time of admission, so we can be guided by what decisions to make in case of emergencies. (ID73)

Treatment escalation plans that were discussed and completed on admission helped to ascertain the patients' wishes before they deteriorated. (ID458)

Thus, respondents ensured end of life planning happened early so that patients had the opportunity to be involved prior to any worsening in their condition.

#### 4.3.3 Providing privacy, comfort and meaning

To maintain patients' privacy and dignity, respondents created makeshift barriers during personal care such as ‘a pillowcase on a drip stand to cover the window whilst I checked the patient's skin’ (ID272) and ‘if we could not find any screens, then we would try and hang bed sheets up’ (ID377).

Respondents also described providing patients with meaningful and comforting items, particularly given visitor restrictions and patients' isolation. These included food, clothes, personal items and often related to the patient's culture and spirituality, such as an ‘electronic Bible and Koran to play passages' (ID533). Respondents encouraged family members to drop off such items (ID334), or staff themselves provided them:

Wherever possible [I] did some shopping for the patients to get items they wanted (ID334)

One key purpose of providing patients with items was to ‘occupy the patient's time’ during isolation (ID373), ‘providing things for them to do once they felt well enough’ (ID340) such as reading materials, activity books and playing cards. Respondents also provided patients with comfort by creating ‘all about me boards’ (ID533) comprised of photos and messages provided by significant others:

The [hospital] provided a link address so relatives could send letters and pictures to [the] patient providing talking opportunities. As nurses we placed the pictures around the bed side (ID229)

### 4.4 Theme 4: Management and leadership

To enable staff to use the strategies described, feel equipped to meet patients’ needs and maintain their own well‐being while doing so, respondents highlighted the importance of management and leadership strategies including education and information provision (constituent theme a) and supporting staff (constituent theme b). These strategies were considered useful in the context of staffs’ lack of knowledge about COVID‐19, fear of catching COVID‐19 and lack of expertise coupled with reduced presence from specialist services.

#### 4.4.1 Education and information provision

Respondents described needing training and upskilling in areas including information technology, non‐verbal communication, mobilizing patients, palliative care, escalation pathways, spiritual beliefs and practices and personal protective equipment.

[We] had some training from palliative care about breaking bad news and having difficult conversations over the telephone, this was very useful. (ID627)

[We] would regularly have class sessions on donning and doffing in order to alleviate staff worries and concerns… [We] explained what personal protective equipment was appropriate for, what procedures, and what classed as aerosol generating procedures. (ID71)

To improve their confidence and ability to answer patients’ questions and address fears around catching COVID‐19, respondents noted the importance of being up to date with changes in the pandemic and the latest guidance/policy. Respondents identified multiple methods of communicating this information in a consistent and timely manner, including the intranet, team meetings, bulletins, whiteboards and specific staff acting as ‘COVID champions’ to disseminate information.

Regular updates and bespoke ward communication strategies were useful in keeping staff up to date regarding changing practices, as well as regular "Huddles" facilitated by the senior team. (ID481)

#### 4.4.2 Supporting staff

Respondents described the need to support the well‐being of staff themselves during the challenges of the COVID‐19 pandemic. This included providing access to in‐house counsellors or psychology teams to provide safe spaces where staff could express their concerns and discuss their experiences:

Some staff that were notably concerned regarding their risks were supported by our in‐house psychology team with regular staff wellbeing sessions… This allowed for discussion and support which helped staff feel more ‘looked after’. (ID274)

I used psychology to talk through fears and develop coping strategies. (ID146)

Respondents reported that regular team briefs/huddles were important in providing staff with information, “regular reassurance” (ID626) and a supportive space to “share experiences and concerns” (ID481), “answer questions” (ID564), and share information about how best to support patients. Nurse leaders also sought to provide staff with space away from the ward and ensure they took regular breaks:

The utilisation of safety huddles and ward safety briefings to identify patients at risk and needs that were required by specific patients. Follow up communication with nursing staff to support psychological and emotional wellbeing. Calm room and safe space identified to allow staff time away to recharge and feel ready to support patients. (ID626)

When I was lead nurse in an area I always made sure the staff I was working with went to [the] staff room for a refreshment if acuity permitted. (ID17)

## 5 DISCUSSION

In this study, we report our analysis of the strategies nurses used for addressing the barriers to providing fundamental nursing care for inpatients with COVID‐19 in terms of four themes: communication behaviours; organization of care; addressing patients’ well‐being and values; management and leadership.

Communication: respondents described a range of patient‐facing adaptations including increasing non‐verbal communication, changing verbal communications, increased explanation, checking of understanding and clarifying the nurse's identity. Also reported were regular, scheduled, documented and often password‐protected virtual contact with patients' significant others, patient information visible from outside the patient's room and clear records of patients’ needs and care actions.

Organization: respondents reported reorganizing care including clustering interventions into one room visit, taking all potentially required items into the room, ensuring sufficient stock, encouraging patient self‐care, particularly to address mobility needs and teamwork strategies, such as ‘runners’ to collect supplies, and highlighted the importance of scheduled interdisciplinary input.

Addressing patients’ well‐being and values: this theme included supporting patients’ mental health by spending time with them, providing things normally furnished by their significant others, such as items to comfort them, occupy them and support their spirituality, ensuring access to counsellors, psychologists and chaplains, obtaining information about patients’ values early in admission, to ensure these could be respected should people's condition suddenly deteriorate. Respondents also reported extra efforts during personal care to ensure privacy.

The management and leadership theme included training in areas such as information technology and updates to changes in the pandemic and the latest guidance. Respondents described supporting staff through access to counsellors or psychologists, team huddles encompassing reassurance, and a safe space and regular breaks as methods for supporting staff well‐being.

Our results make a substantial contribution to the disappointingly scant existing empirical literature on fundamental nursing care in a pandemic, reviewed in (Whear et al., 2022). Of the 12 previous qualitative studies in this area, most are very small and report nurses’ experiences of care, not their adaptive strategies. Unlike our study, in previous studies, any suggestions for care strategies are mostly author generated, rather than being based on nurse data directly (e.g. Corley et al., 2010; Kang et al., 2018; Shih et al., 2007). Our study is the largest to date that has sought data directly from nurses on strategies to adapt their care. Our study is also the first in this area that explicitly uses the Fundamentals of Care framework (Kitson et al., 2010) to structure data collection and analysis.

Nonetheless, our respondents’ adaptive strategies encompass those identified previously, including in non‐research‐based commentaries, for example using photos on personal protective equipment and exaggerating non‐verbal communication (Brown‐Johnson et al., 2020; Pettis, 2020); writing information down and using whiteboards (Bagnasco et al., 2020; Fedele, 2020); using technology for daily contact with patients’ significant others, including providing phones to patients (Chan et al., 2006; Fan et al., 2020; Neville, 2020; Pettis, 2020; Tsai et al., 2020) and providing clear explanations to patients (Hart et al., 2020). Similarly, our respondents and this literature identify reorganizing care by working in pairs (Liu & Liehr, 2009), using ‘runners’ (Newby et al., 2020) and clustering interventions (Kuntz et al., 2020).

For the same reasons as above, our data also confirm the literature on pandemic nursing leadership. The management and leadership strategies identified by our respondents concur with those cited elsewhere, in terms of supporting staffs’ knowledge, confidence and well‐being. Strategies mentioned in other literature include providing staff training (Holmgren et al., 2019; Pahuja & Wojcikewych, 2021), meetings or huddles (Cathcart, 2020; Corley et al., 2010; Martland & Huffines, 2020), psychological support (Bagnasco et al., 2020; Cathcart, 2020; Hofmeyer et al., 2020a; Hofmeyer et al., 2020b; Martland & Huffines, 2020; Morley et al., 2020) and the provision of timely pandemic information and guidelines (Adams, 2020; Kang et al., 2018; Liu & Liehr, 2009; Martland & Huffines, 2020).

While our respondents’ strategies, in emphasizing education and support around the *content* of leadership, strengthen the findings from much smaller studies, one data‐based qualitative study of Ebola Virus Disease (EVD) nursing also includes recommendations for management [leadership] style, to include ‘listening, professionalism, calmness, experience, structural ability, effectiveness, encouragement, empathy, social competence, support, information and objectivity’ (Holmgren et al., 2019), p829. The only study we are aware of to directly include data from a large cohort of leaders themselves—involved in the Taiwanese SARS epidemic (Shih et al., 2007)—also stressed additional elements of emotional intelligence, socio‐political and analytical leadership qualities. While our data only confirm the desirability for ground‐level supportive leadership behaviours of senior nurses, it also seems clear to us that macro‐level behaviours noted by Shih and colleagues, where nurses are seen to be politically adept and authoritatively leading the pandemic response in their interaction with mainstream and social media, are also required.

### 5.1 Limitations

One limitation of this study is our convenience sampling frame, with a sample size determined by the period of time the survey was open, which was in turn constrained by the need to gather information quickly to design our ‘COVID‐NURSE’ intervention (Richards et al., 2021). We also potentially experienced respondent fatigue, whereby the number of respondents providing data generally reduced towards the end of the survey, although we do not know if the small number of narrative responses to individual items is a product of fatigue or that respondents genuinely did not have any strategies to contribute (Lavrakas, 2008). However, in all items/each sub‐category of fundamental care we did reach data saturation, at which point data from additional respondents were no longer providing additional clarity or insight; thus, our sample size in and across each sub‐category of care could be considered adequate as well as appropriate as our eligibility criteria ensured respondents were experts in the area of interest (Morse, 1995; Morse, 2015).

It may have been beneficial to include Allied Health Professionals in our sample; this may have provided additional insights, particularly in relation to our finding that input from Allied Health Professionals was considered crucial by our respondents for meeting patients’ fundamental care needs. However, time constraints for developing our intervention meant that we were unable to send out a second round of the survey once we had gathered this insight. Finally, given the proportions of different staff completing the survey, our results largely represent the views of registered nurses rather than non‐registered members of the nursing workforce who may have held differing views or offered additional insights.

## 6 CONCLUSIONS

In our survey of nurses caring for inpatients with COVID‐19, we identified four themes to understand respondents’ strategies when overcoming barriers to meeting patients’ fundamental care needs: communication behaviours; organization of care; addressing patients’ well‐being and values; management and leadership. These findings support the Fundamentals of Care model, where communication is at the heart of all good nursing care, establishing the platform for compassionate and collaborative transactional care to address patients’ physical and psychosocial needs (Feo et al., 2018; Kitson et al., 2010). Many of the strategies identified by our respondents may help to address the negative experiences reported by patients and significant others, including improving communication with staff, providing an increased level of information, ensuring sufficient equipment is available, improving patients’ mobility, supporting patients’ well‐being in the face of decreased social interaction and keeping significant others up to date (Healthwatch, 2020; Shaban et al., 2020).

In particular, we draw attention to the need for management and leadership. It is here where nursing, particularly fundamental care nursing, has much to gain. Leadership on the ground is required to ensure nurses are trained, upskilled and up to date by the timely provision of pandemic information. Good leaders provide psychological support, for example using team huddles and ensuring that nurses’ needs for breaks and self‐care are identified and acted on. However, although not an element of our thematic analyses, nurses can learn from the pandemic experiences of others (Shih et al., 2007) in using the disruptive potential of the COVID‐19 pandemic to advance the value of nursing, particularly those fundamental care behaviours included in the FoC model (Feo et al., 2018, Kitson et al., 2010), by engaging forcefully and visibly with political, policy and media actors.

Although our data were collected from a specific nursing context—inpatient care for patients with COVID‐19 who were not invasively ventilated‐these strategies are unlikely to be specific to the COVID‐19 pandemic or the hospital environment represented by our respondents. They may, therefore, inform planners devising strategies to deliver nursing care in other environments (such as care homes), other countries and for other pandemics globally.

Unfortunately, there is currently extremely limited evidence on the effectiveness of any of the identified strategies. Few studies focus on patient experience or outcomes. Although many COVID‐19 resources now exist, for example staff well‐being (The Kings Fund, 2021), infection control (Gould & Purssell, 2021) and oxygen management (Messer et al., 2021), fundamental nursing care has not been the focus of guidelines (Whear et al., 2022). To prepare for the continuation of the COVID‐19 pandemic and future pandemics, the effectiveness of these strategies for meeting patients’ fundamental care must be evaluated. This research need will enable evidence‐based care to be provided in future and provide guidance for educators, clinicians, managers, leaders and policymakers on potentially useful strategies for meeting patients’ fundamental care needs in pandemics and other situations where patients are nursed in isolation. Now that we have a large amount of observational data driven by the FoC model, experimental research is our research community's next step (Richards, 2020). Consequently, we have incorporated these strategies into a fundamental nursing care clinical protocol that we are currently evaluating in a cluster randomized controlled trial (Richards et al., 2021). This should be only the first step in rigorously testing the FoC model in multiple different settings, cultures and contexts.

## CONFLICT OF INTEREST

No conflict of interest has been declared by the authors.

### PEER REVIEW

The peer review history for this article is available at https://publons.com/publon/10.1111/jan.15261.

## Supporting information


Supplementary Material

## Data Availability

Research data are not shared due to the sensitivity of qualitative narrative data

## References

[jan15261-bib-0001] Adams, C. (2020). Goals of Care in a Pandemic: Our experience and recommendations. Journal of Pain and Symptom Management, 60, e15–e17. 10.1016/j.jpainsymman.2020.03.018 PMC7270987 32240752

[jan15261-bib-0002] Aiken, L. H. , Sloane, D. , Griffiths, P. , Rafferty, A. M. , Bruyneel, L. , McHugh, M. , Maier, C. B. , Moreno‐Casbas, T. , Ball, J. E. , Ausserhofer, D. , Sermeus, W. , & Consortium, R. C. (2017). Nursing skill mix in European hospitals: Cross‐sectional study of the association with mortality, patient ratings, and quality of care. Bmj Quality & Safety, 26, 559–568. 10.1136/bmjqs-2016-005567 PMC5477662 28626086

[jan15261-bib-0003] Aiken, L. H. , Sloane, D. M. , Bruyneel, L. , Van den Heede, K. , Griffiths, P. , Busse, R. , Diomidous, M. , Kinnunen, J. , Kozka, M. , Lesaffre, E. , McHugh, M. D. , Moreno‐Casbas, M. T. , Rafferty, A. M. , Schwendimann, R. , Scott, P. A. , Tishelman, C. , Van Achterberg, T. , & Sermeus, W. (2014). Nurse staffing and education and hospital mortality in nine European countries: A retrospective observational study. Lancet, 383, 1824–1830. 24581683 10.1016/S0140-6736(13)62631-8 PMC4035380

[jan15261-bib-0004] Ausserhofer, D. , Zander, B. , Busse, R. , Schubert, M. , De Geest, S. , Rafferty, A. M. , Ball, J. , Scott, A. , Kinnunen, J. , Heinen, M. , Sjetne, I. S. , Moreno‐Casbas, T. , Kozka, M. , Lindqvist, R. , Diomidous, M. , Bruyneel, L. , Sermeus, W. , Aiken, L. H. , Schwendimann, R. , & Consortium, R. C. (2014). Prevalence, patterns and predictors of nursing care left undone in European hospitals: Results from the multicountry cross‐sectional RN4CAST study. BMJ Quality and Safety, 23, 126–135. 10.1136/bmjqs-2013-002318 24214796

[jan15261-bib-0005] Bagnasco, A. , Zanini, M. , Hayter, M. , Catania, G. , & Sasso, L. (2020). COVID 19‐a message from Italy to the global nursing community. Journal of Advanced Nursing, 76, 2212–2214. 32352175 10.1111/jan.14407 PMC7267658

[jan15261-bib-0006] Ball, J. E. , Bruyneel, L. , Aiken, L. H. , Sermeus, W. , Sloane, D. M. , Rafferty, A. M. , Lindqvist, R. , Tishelman, C. , Griffiths, P. , & Consortium, R. N. C. (2018). Post‐operative mortality, missed care and nurse staffing in nine countries: A cross‐sectional study. International Journal of Nursing Studies, 78, 10–15. 28844649 10.1016/j.ijnurstu.2017.08.004 PMC5826775

[jan15261-bib-0007] Ball, J. E. , Griffiths, P. , Rafferty, A. M. , Lindqvist, R. , Murrells, T. , & Tishelman, C. (2016). A cross‐sectional study of 'care left undone' on nursing shifts in hospitals. Journal of Advanced Nursing, 72, 2086–2097. 27095463 10.1111/jan.12976

[jan15261-bib-0008] Barbour, R. S. (2001). Checklists for improving rigour in qualitative research: A case of the tail wagging the dog? BMJ, 322, 1115–1117. 11337448 10.1136/bmj.322.7294.1115 PMC1120242

[jan15261-bib-0009] Black, N. , Varaganum, M. , & Hutchings, A. (2014). Relationship between patient reported experience (PREMs) and patient reported outcomes (PROMs) in elective surgery. BMJ Quality and Safety, 23, 534–542. 10.1136/bmjqs-2013-002707 24508681

[jan15261-bib-0010] Brown‐Johnson, C. , Vilendrer, S. , Heffernan, M. B. , Winter, S. , Khong, T. , Reidy, J. , & Asch, S. M. (2020). PPE portraits‐a way to humanize personal protective equipment. Journal of General Internal Medicine, 35, 2240–2242. 32410125 10.1007/s11606-020-05875-2 PMC7224350

[jan15261-bib-0011] Bruyneel, L. , Li, B. , Ausserhofer, D. , Lesaffre, E. , Dumitrescu, I. , Smith, H. L. , Sloane, D. M. , Aiken, L. H. , & Sermeus, W. (2015). Organization of Hospital Nursing, provision of nursing Care, and patient experiences with Care in Europe. Medical Care Research and Review, 72, 643–664. 26062612 10.1177/1077558715589188 PMC4631674

[jan15261-bib-0012] Cathcart, E. B. (2020). The new nurse manager survival guide, part II. Nursing Management, 51, 17–20. 10.1097/01.NUMA.0000662704.97080.df PMC7258746 32379170

[jan15261-bib-0013] Chan, E. A. , Chung, J. W. , Wong, T. K. , & Yang, J. C. (2006). An evaluation of nursing practice models in the context of the severe acute respiratory syndrome epidemic in Hong Kong: A preliminary study. Journal of Clinical Nursing, 15, 661–670. 16684161 10.1111/j.1365-2702.2006.01345.x

[jan15261-bib-0014] Corley, A. , Hammond, N. E. , & Fraser, J. F. (2010). The experiences of health care workers employed in an Australian intensive care unit during the H1N1 influenza pandemic of 2009: A phenomenological study. International Journal of Nursing Studies, 47, 577–585. 20036360 10.1016/j.ijnurstu.2009.11.015 PMC7125717

[jan15261-bib-0015] Darzi, A. (2008). Quality and the NHS next stage review. Lancet, 371, 1563–1564. 18468532 10.1016/S0140-6736(08)60672-8

[jan15261-bib-0016] Doyle, C. , Lennox, L. , & Bell, D. (2013). A systematic review of evidence on the links between patient experience and clinical safety and effectiveness. BMJ Open, 3, e001570. 10.1136/bmjopen-2012-001570 PMC3549241 23293244

[jan15261-bib-0017] Fan, P. E. M. , Aloweni, F. , Lim, S. H. , Ang, S. Y. , Perera, K. , Quek, A. H. , Quek, H. K. S. , & Ayre, T. C. (2020). Needs and concerns of patients in isolation care units—learnings from COVID‐19: A reflection. World Journal of Clinical Cases, 8, 1763–1766. 32518768 10.12998/wjcc.v8.i10.1763 PMC7262715

[jan15261-bib-0018] Fedele, R. (2020). It's surreal': Nursing during the COVID‐19 pandemic. Australian Nursing and Midwifery Journal, 26, 6–7. https://anmj.org.au/its‐surreal‐nursing‐during‐the‐covid‐19‐pandemic/

[jan15261-bib-0019] Feo, R. , Conroy, T. , Jangland, E. , Muntlin Athlin, A. , Brovall, M. , Parr, J. , Blomberg, K. , & Kitson, A. (2018). Towards a standardised definition for fundamental care: A modified Delphi study. Journal of Clinical Nursing, 27, 2285–2299. 29278437 10.1111/jocn.14247

[jan15261-bib-0020] Gould, D. , & Purssell, E. (2021). RCN independent review of guidelines for the prevention and control of Covid‐19 in health care settings in the United Kingdom: Evaluation and messages for future infection‐related emergency planning. Royal College of Nursing.

[jan15261-bib-0021] Graham, C. , Käsbauer, S. , Cooper, R. , King, J. , Sizmur, S. , Jenkinson, C. , & Kelly, L. (2018). An evaluation of a near real‐time survey for improving patients' experiences of the relational aspects of care: A mixed‐methods evaluation. Southampton. 29595933

[jan15261-bib-0022] Griffiths, P. , Recio‐Saucedo, A. , Dall'ora, C. , Briggs, J. , Maruotti, A. , Meredith, P. , Smith, G. B. , Ball, J. , & Missed Care Study, G. (2018). The association between nurse staffing and omissions in nursing care: A systematic review. Journal of Advanced Nursing, 74, 1474–1487. 29517813 10.1111/jan.13564 PMC6033178

[jan15261-bib-0023] Hart, J. L. , Turnbull, A. E. , Oppenheim, I. M. , & Courtright, K. R. (2020). Family‐centered Care during the COVID‐19 era. Journal of Pain and Symptom Management, 60, e93–e97. 32333961 10.1016/j.jpainsymman.2020.04.017 PMC7175858

[jan15261-bib-0024] Healthwatch . 2020. 590 people's stories of leaving hospital during COVID‐19 [Online]. Available: https://www.healthwatch.co.uk/sites/healthwatch.co.uk/files/20201026%20Peoples%20experiences%20of%20leaving%20hospital%20during%20COVID‐19_0.pdf [Accessed 08/12/2021 2021].

[jan15261-bib-0025] Hofmeyer, A. , Taylor, R. , & Kennedy, K. (2020a). Fostering compassion and reducing burnout: How can health system leaders respond in the Covid‐19 pandemic and beyond? Nurse Education Today, 94, 104502. 10.1016/j.nedt.2020.104502 32980180 PMC7295512

[jan15261-bib-0026] Hofmeyer, A. , Taylor, R. , & Kennedy, K. (2020b). Knowledge for nurses to better care for themselves so they can better care for others during the Covid‐19 pandemic and beyond. Nurse Education Today, 94, 104503. 10.1016/j.nedt.2020.104503 32980179 PMC7295457

[jan15261-bib-0027] Holmgren, J. , Paillard‐BORG, S. , Saaristo, P. , & Von Strauss, E. (2019). Nurses' experiences of health concerns, teamwork, leadership and knowledge transfer during an Ebola outbreak in West Africa. Nursing Open, 6, 824–833. 31367405 10.1002/nop2.258 PMC6650671

[jan15261-bib-0028] Houghton, C. , Casey, D. , Shaw, D. , & Murphy, K. (2013). Rigour in qualitative case‐study research. Nurse Researcher, 20, 12–17. 10.7748/nr2013.03.20.4.12.e326 23520707

[jan15261-bib-0029] International Learning Collaborative . 2019. The Fundamentals of Care [Online]. Available: https://intlearningcollab.org/mission/the‐fundamentals‐of‐care/#physical [Accessed 08/12/2021 2021].

[jan15261-bib-0030] Kalisch, B. J. (2006). Missed nursing care: A qualitative study. Journal of Nursing Care Quality, 21, 306–313. 16985399 10.1097/00001786-200610000-00006

[jan15261-bib-0031] Kalisch, B. J. , Landstrom, G. L. , & Hinshaw, A. S. (2009). Missed nursing care: A concept analysis. Journal of Advanced Nursing, 65, 1509–1517. 19456994 10.1111/j.1365-2648.2009.05027.x

[jan15261-bib-0032] Kalisch, B. J. , Xie, B. , & Dabney, B. W. (2014). Patient‐reported missed nursing care correlated with adverse events. American Journal of Medical Quality, 29, 415–422. 24006031 10.1177/1062860613501715

[jan15261-bib-0033] Kang, H. S. , Son, Y. D. , Chae, S. M. , & Corte, C. (2018). Working experiences of nurses during the Middle East respiratory syndrome outbreak. International Journal of Nursing Practice, 24, e12664. 29851209 10.1111/ijn.12664 PMC7165521

[jan15261-bib-0034] Kitson, A. , Conroy, T. , Wengstrom, Y. , Profetto‐McGrath, J. , & Robertson‐Malt, S. (2010). Defining the fundamentals of care. International Journal of Nursing Practice, 16(4), 423–434. 20649678 10.1111/j.1440-172X.2010.01861.x

[jan15261-bib-0035] Knottnerus, A. , & Tugwell, P. (2008). STROBE—A checklist to STrengthen the reporting of OBservational studies in epidemiology. Journal of Clinical Epidemiology, 61, 323. 18313555 10.1016/j.jclinepi.2007.11.006

[jan15261-bib-0036] Kuntz, J. G. , Kavalieratos, D. , Esper, G. J. , Ogbu, N., Jr. , Mitchell, J. , Ellis, C. M. , & Quest, T. (2020). Feasibility and acceptability of inpatient palliative Care E‐family meetings during COVID‐19 pandemic. Journal of Pain and Symptom Management, 60, e28–e32. 10.1016/j.jpainsymman.2020.06.001 PMC7272163 32505643

[jan15261-bib-0037] Lavrakas, P. J. (2008). Encyclopedia of survey research methods. Sage Publications.

[jan15261-bib-0038] Liu, H. , & Liehr, P. (2009). Instructive messages from Chinese nurses' stories of caring for SARS patients. Journal of Clinical Nursing, 18, 2880–2887. 19747256 10.1111/j.1365-2702.2009.02857.x

[jan15261-bib-0039] Maben, J. , & Bridges, J. (2020). Covid‐19: Supporting nurses' psychological and mental health. Journal of Clinical Nursing, 29, 2742–2750. 32320509 10.1111/jocn.15307 PMC7264545

[jan15261-bib-0040] Martland, A. M. & Huffines, M. 2020. Surge priority planning COVID‐19: Critical care staffing and nursing considerations [online]. Chest. Available: https://www.chestnet.org/resources/surge‐priority‐planning‐covid‐19‐critical‐care‐staffing‐and‐nursing‐considerations [Accessed 08/12/2021 2021].

[jan15261-bib-0041] Messer, B. , Antoine‐Pitterson, P. , Blundell, A. , Burns, G. , Davies, M. , Moonesinghe, R. , Uylsteke, A. , Webb, S. , & Wood, S. (2021). BTS/ICS guidance: Respiratory care in patients with acute Hypoxaemic respiratory failure associated with COVID‐19 V2. British Thoracic Society and the Intensive Care Society.

[jan15261-bib-0042] Microsoft Coproration . (2013). Microsoft Excel. Microsoft Corporation.

[jan15261-bib-0043] Miles, M. B. , & Huberman, A. M. (1994). Qualitative data analysis: An expanded sourcebook. Sage.

[jan15261-bib-0044] Miles, M. B. , Huberman, A. M. , & Saldana, J. (2014). Qualitative data analysis: A methods sourcebook. Sage.

[jan15261-bib-0045] Morley, G. , Sese, D. , Rajendram, P. , & Horsburgh, C. C. (2020). Addressing caregiver moral distress during the COVID‐19 pandemic. Cleveland Clinic Journal of Medicine. 10.3949/ccjm.87a.ccc047 32518134

[jan15261-bib-0046] Morse, J. M. (1995). The significance of saturation. In The significance of saturation. Thousand Oaks.

[jan15261-bib-0047] Morse, J. M. (2015). Data were saturated …. Qualitative Health Research, 25(5), 587–588. 25829508 10.1177/1049732315576699

[jan15261-bib-0048] Murrells, T. , Robert, G. , Adams, M. , Morrow, E. , & Maben, J. (2013). Measuring relational aspects of hospital care in England with the 'Patient evaluation of emotional Care during Hospitalisation' (PEECH) survey questionnaire. BMJ Open, 3, e002211. 10.1136/bmjopen-2012-002211 PMC3563120 23370012

[jan15261-bib-0049] Neville, T. H. (2020). COVID‐19: A time for creative compassion. Journal of Palliative Medicine, 23, 990–991. 32380916 10.1089/jpm.2020.0242

[jan15261-bib-0050] Newby, J. C. , Mabry, M. C. , Carlisle, B. A. , Olson, D. M. , & Lane, B. E. (2020). Reflections on nursing ingenuity during the COVID‐19 pandemic. Journal of Neuroscience Nursing, 52, E13–E16. 10.1097/JNN.0000000000000525 PMC7172973 32221059

[jan15261-bib-0051] Office for National Statistics . 2019. Research report on population estimates by ethnic group and religion [Online]. Available: https://www.ons.gov.uk/peoplepopulationandcommunity/populationandmigration/populationestimates/articles/researchreportonpopulationestimatesbyethnicgroupandreligion/2019‐12‐04 [Accessed 04/08/2020 2020].

[jan15261-bib-0052] Pahuja, M. , & Wojcikewych, D. (2021). Systems barriers to assessment and treatment of COVID‐19 positive patients at the end of life. Journal of Palliative Medicine, 24, 302–304. 32384004 10.1089/jpm.2020.0190

[jan15261-bib-0053] Pettis, J. (2020). Creativity and compassion: NICHE nurses address older Adult's Care needs during the COVID‐19 pandemic. Geriatric Nursing, 41, 509–510.

[jan15261-bib-0054] Pope, C. , & Mays, N. (2006). Qualitative research in health care. Blackwell Publishing.

[jan15261-bib-0055] Qualtrics . (2020). Qualtrics survey software. Qualtrics.

[jan15261-bib-0056] Recio‐Saucedo, A. , Dall'ora, C. , Maruotti, A. , Ball, J. , Briggs, J. , Meredith, P. , Redfern, O. C. , Kovacs, C. , Prytherch, D. , Smith, G. B. , & Griffiths, P. (2018). What impact does nursing care left undone have on patient outcomes? Review of the literature. Journal of Clinical Nursing, 27, 2248–2259. 28859254 10.1111/jocn.14058 PMC6001747

[jan15261-bib-0057] Richards, D. A. (2020). Observational research on fundamental nursing care: Enough already! Journal of Clinical Nursing, 29, 1765–1767. 32057142 10.1111/jocn.15203 PMC7319421

[jan15261-bib-0058] Richards, D. A. , Sugg, H. V. , Cockcroft, E. , Cooper, J. , Cruickshank, S. , Doris, F. , Hulme, C. , Logan, P. , Iles‐Smith, H. , Melendez‐Torres, G. J. , Rafferty, A. M. , Reed, N. , Russell, A. M. , Shepherd, M. , Singh, S. J. , Thompson Coon, J. , Tooze, S. , Wootton, S. , Abbott, R. , … Romanczuk, L. (2021). COVID‐NURSE: Evaluation of a fundamental nursing care protocol compared with care as usual on experience of care for noninvasively ventilated patients in hospital with the SARS‐CoV‐2 virus‐protocol for a cluster randomised controlled trial. BMJ Open, 11, e046436. 10.1136/bmjopen-2020-046436 PMC8159671 34039574

[jan15261-bib-0059] Ritchie, J. , Lewis, J. , Nicholls, C. M. , & Ormston, R. (2013). Qualitative research practice: A guide for social science students and researchers (2nd ed.). Sage.

[jan15261-bib-0060] Shaban, R. Z. , Nahidi, S. , Sotomayor‐Castillo, C. , Li, C. , Gilroy, N. , O'Sullivan, M. V. N. , Sorrell, T. C. , White, E. , Hackett, K. , & Bag, S. (2020). SARS‐CoV‐2 infection and COVID‐19: The lived experience and perceptions of patients in isolation and care in an Australian healthcare setting. American Journal of Infection Control, 48, 1445–1450. 32890549 10.1016/j.ajic.2020.08.032 PMC7466942

[jan15261-bib-0061] Shih, F. J. , Gau, M. L. , Kao, C. C. , Yang, C. Y. , Lin, Y. S. , Liao, Y. C. , & Sheu, S. J. (2007). Dying and caring on the edge: Taiwan's surviving nurses' reflections on taking care of patients with severe acute respiratory syndrome. Applied Nursing Research, 20, 171–180. 17996803 10.1016/j.apnr.2006.08.007 PMC7127079

[jan15261-bib-0062] Spencer, L. , Ritchie, J. , O'Connor, W. , Morrell, G. , & Ormston, R. (2014). Analysis in practice. In J. Ritchie , J. Lewis , C. McNaughton Nicholls , & R. Ormston (Eds.), Qualitative research practice. Sage.

[jan15261-bib-0063] Sugg, H. V. R. , Russell, A. M. , Morgan, L. M. , Iles‐Smith, H. , Richards, D. A. , Morley, N. , Burnett, S. , Cockcroft, E. J. , Thompson Coon, J. , Cruickshank, S. , Doris, F. E. , Hunt, H. A. , Kent, M. , Logan, P. A. , Rafferty, A. M. , Shepherd, M. H. , Singh, S. J. , Tooze, S. J. , & Whear, R. (2021). Fundamental nursing care in patients with the SARS‐CoV‐2 virus: Results from the 'COVID‐NURSE' mixed methods survey into nurses' experiences of missed care and barriers to care. BMC Nursing, 20, 215. 34724949 10.1186/s12912-021-00746-5 PMC8558545

[jan15261-bib-0064] The Kings Fund . 2021. Leading through Covid‐19: supporting health and care leaders in unprecedented times [Online]. Available: https://www.kingsfund.org.uk/projects/leading‐through‐covid‐19 [Accessed 08/12/2021 2021].

[jan15261-bib-0065] Thorne, S. E. (2000). Data analysis in qualitative research. Evidence Based Nursing, 3, 68–70.

[jan15261-bib-0066] Tong, A. , Sainsbury, P. , & Craig, J. (2007). Consolidated criteria for reporting qualitative research (COREQ): A 32‐item checklist for interviews and focus groups. International Journal for Quality in Health Care, 19, 349–357. 17872937 10.1093/intqhc/mzm042

[jan15261-bib-0067] Torjesen, I. (2021). Covid‐19: When to start invasive ventilation is "the million dollar question". BMJ, 372, n121. 33446505 10.1136/bmj.n121

[jan15261-bib-0068] Tsai, M. J. , Tsai, W. T. , Pan, H. S. , Hu, C. K. , Chou, A. N. , Juang, S. F. , Huang, M. K. , & Hou, M. F. (2020). Deployment of information technology to facilitate patient care in the isolation ward during COVID‐19 pandemic. Journal of the American Medical Informatics Association, 27, 1819–1820. 32516374 10.1093/jamia/ocaa126 PMC7314018

[jan15261-bib-0069] University of Cardiff . The ICON Study [Online]. Available: https://www.cardiff.ac.uk/healthcare‐sciences/research/projects/the‐icon‐study‐an‐ongoing‐study‐of‐the‐impact‐of‐covid‐19‐on‐the‐nursing‐workforce [Accessed 08/12/2021 2021].

[jan15261-bib-0070] Whear, R. , Abbott, R. A. , Bethel, A. , Richards, D. A. , Garside, R. , Cockcroft, E. , Iles‐Smith, H. , Logan, P. A. , Rafferty, A. M. , Shepherd, M. , Sugg, H. V. R. , Russell, A. M. , Cruickshank, S. , Tooze, S. , Melendez‐Torres, G. J. , & Thompson Coon, J. (2022). Impact of COVID‐19 and other infectious conditions requiring isolation on the provision of and adaptations to fundamental nursing care in hospital in terms of overall patient experience, care quality, functional ability, and treatment outcomes: Systematic review. Journal of Advanced Nursing, 78, 78–108. 34554585 10.1111/jan.15047 PMC8657334

[jan15261-bib-0071] Yardley, L. (2017). Demonstrating the validity of qualitative research. The Journal of Positive Psychology, 12, 295–296.

